# Factors Affecting Urinary tt-Muconic Acid Detection among Benzene Exposed Workers at Gasoline Stations

**DOI:** 10.3390/ijerph16214209

**Published:** 2019-10-30

**Authors:** Sunisa Chaiklieng, Pornnapa Suggaravetsiri, Norbert Kaminski, Herman Autrup

**Affiliations:** 1 Department of Environmental Health, Occupational Health and Safety, Faculty of Public Health, Khon Kaen University, Khon Kaen 40002, Thailand; 2 Department of Epidemiology and Biostatistics, Faculty of Public Health, Khon Kaen University, Khon Kaen 40002, Thailand; porsug@kku.ac.th; 3 Institute for Integrative Toxicology, Michigan State University, East Lansing, MI 48224, USA; kamins11@msu.edu; 4 Institute of Public Health, Aarhus University, 80000 Aarhus, Denmark; ha@ph.au.dk

**Keywords:** benzene, biomarker, exposure assessment, risk

## Abstract

Trans, trans-muconic acid (tt-MA) is a metabolite that is widely used as a biomarker to identify low exposure to benzene, a human carcinogen. This study aimed to investigate occupational factors related to the urinary tt-MA detection of benzene exposed workers in gasoline stations. Spot urine samples were collected and analyzed for tt-MA using a high performance liquid chromatography. Additional data were collected via subject interviews using a structured questionnaire. The personal benzene concentration was measured and analyzed by gas chromatography with a flame ionization detector. Results showed that, among the 170 workers, tt-MA was detected in 24.7% of workers and the concentration ranged from 23.0 to 1127.8 µg/g creatinine. Over 25% of those detections possessing tt-MA exceeding the recommended 500 µg/g creatinine was safe. A multiple logistic regression analysis identified that factors significantly associated with the detectable tt-MA were having no other part-time jobs (ORadj = 4.2), personal benzene concentrations of 0.05 ppm or higher (ORadj = 10.3), close to fuel nozzle during refuelling (ORadj = 93.7), and no job training (ORadj = 2.74). Safety training is recommended for those tt-MA detected workers or under a reference benzene concentration of 0.05 ppm or higher. The proposed reference of occupational action level to benzene exposure is 0.05 ppm and compliance could be assessed tt-MA for biomonitoring of those benzene exposed workers.

## 1. Introduction

Animal and human data show a strong association between benzene exposure and leukemia, hence benzene was classified as a group I human carcinogen by the International Agency of Research for Cancer, or IARC [1]. In 2008, Thailand was identified as one of forty countries in the world with a high incidence of leukemia [2]. The Bureau of Epidemiology in Thailand reported that of all cases of organic solvent toxicity, benzene was the most common causative agent with the prevalence being 15% of all cases [3]. The incidence rate of benzene toxicity has shown an increasing trend between 1994 to 2012 [4] with the main cause attributed to occupational exposure among workers in the manufacturing of basic chemical solvents and refined petroleum products [2,5]. Therefore, health surveillance could play an important role in protecting workers in the petrochemical industry.

The identification of reliable biomarkers is one tool for the effective health surveillance of workers in detecting occupational exposure to benzene. S-phenylmercapturic acid (S-PMA) and trans, trans-muconic acid (tt-MA) have been recommended as biomarkers of benzene exposure. Both metabolites can be detected in the urine of workers collected at the end of the work shift, and tt-MA has a sensitivity to be detected at benzene exposure below 1 ppm (part per million) [6]. However, biological monitoring for occupational benzene exposure tends to be confounded by several factors that may influence the tt-MA level, a biomarker of benzene exposure such as eating food-contained sorbic acid, off work environmental exposure, individual susceptibility, and working behaviors [7]. Cigarette smoking is an important source of exposure to benzene [8], which would be the direct internal dose of benzene inhaled to elevate levels of the tt-MA metabolite as a consequence.

To our knowledge, no prior studies have reported on the specific work characteristics and behaviors related to safety practices that may influence benzene exposure. Low benzene concentrations in the ambient and inhaled air of workers at gas stations (<0.1 ppm) have been previously reported [9]. Interestingly, it has also been shown that these workers had an increased risk of cancer after long-term benzene exposure [10,11]. According to several studies where tt-MA was measured in the context of occupational exposure to benzene, tt-MA was found to be a reliable determinant of benzene exposure, especially for the chronic low level benzene exposure of gasoline station workers [12,13]. Based on the above, biomonitoring benzene exposure, by assessing urinary tt-MA levels in combination with the air concentration of benzene in the occupational setting, should be strongly considered. The latter could be effectively accomplished through the use of an individual personal air sampling device [7].

Presently, there is limited information on the factors that may influence the urinary tt-MA levels of workers at gasoline stations. Therefore, the objective of this study was to identify potential factors that could influence the level of the biomarker for an internal dose of benzene exposure (i.e., urinary tt-MA). The outcomes that this investigation provides are important considerations when using tt-MA urine levels as a measure of the biomarker of internal dose of benzene exposure, in order to confirm that of the validity of tt-MA as a biomarker for occupational exposure to benzene at low doses.

## 2. Materials and Methods

### 2.1. Study Population and Sample Size

This study was designed as a cross-sectional analytic study to investigate the factors associated with tt-MA as a biomarker for benzene exposure. There were 98 gasoline stations in the city of Muang Khon Kaen, the capital city of Khon Kaen Province, Thailand and a total of 686 workers at these gasoline stations [14]. The participants recruited for this study worked at different gasoline stations in the city of Muang Khon Kaen. The gasoline stations were categorized into three zones: ‘urban’, ‘suburban’, and ‘rural’ based upon their geographical location. The gasoline stations in the urban zone were those located in the Nai Muang subdistrict of the city of Khon Kaen and where the majority of the residents’ occupation was not in agriculture; and the suburban gasoline stations were located around the Nai Muang subdistrict and near the main highway, Mittraparp Road, which connects the provincial city of Khon Kaen to the capital city of Bangkok. The rural gasoline stations were those located outside the Nai Muang subdistrict and where the majority of the residents worked in agriculture.

The sample size was calculated to detect the factor associated with the tt-MA biomarker of benzene exposure using an approach of a simple method of calculation in a cross-sectional analytic study for logistic regression analysis [15]. The independent variable of interest was the benzene concentrations found in work areas. The reference rate of a worker exposed to benzene via the tt-MA biomarker level detection among gasoline workers working in areas with a benzene concentration below 1 ppm was 19% (P0 = 0.19) [11]. The desired level of confidence was 95% (α = 0.05) and the power of 80% (ß = 0.20). Therefore, the minimum requirement of sample size in the simple logistic regression was 80 gasoline workers. For multivariate analysis, the sample size was further adjusted for reproducibility results following Hsieh 1998’s optimized formula [15]. At ∂ = 0.5, the required number of subjects was 150 workers. All subjects who met the inclusion criteria: (1) at least 18 years of age, (2) work at least 8 h per day, (3) employed at the gasoline station for more than six months, and (4) a non-smoker, were included by systematic random sampling. The condition of a good distribution of participants representative of the station and workplace zone (i.e., urban, suburban, and rural zone) was considered and 10% of that required number was added for the different job functions such as cashiers (*n* = 15). The final sample size was 170 of gasoline station workers included in the study. Alcohol drinking and passive smoking were prohibited for 24 h before urine sample collection from the included subjects. Those factors were controlled in order to confirm the validity of tt-MA for the biomonitoring of occupational exposure to low benzene concentrations.

### 2.2. Data Collection and Analysis

The structured questionnaire was developed based upon a literature review, following notification of the Ministry of Industry, Thailand for the health risk assessment on hazardous chemical exposure in an occupational setting (B.E.2555) [16]. The questionnaire was approved by the Ministry of Industry, Thailand and then tested and validated prior to use for data collection.

This questionnaire included three parts: the first part queried personal information, the second part queried work characteristics and environment, and the third part queried safety practices and job training.

The benzene concentration was measured by personal air monitoring. Each air sample was collected by using a coconut charcoal sorbent tube that was fixed in the breathing zone for each worker that was connected to an active personal pump. The personal air sampling was carried out through 8 h of working period. The samples were analyzed by gas chromatography with a flame ionization detector (GC-FID). Methods of air monitoring and analysis of benzene concentration followed the National Institute for Occupational Safety and Health (NIOSH) standard method number 1501 [17]. Results from the authors’ previous study showed that the concentration range of inhaled benzene by personal sampling was from 0.03 ppb to 65.71 ppb; however, the range for benzene in ambient air by area sampling was from 7.50 to 50.00 ppb [9]. Therefore, the benzene concentration of 50 ppb, which was 50% of the occupational exposure limit (OEL) of exposure concentration for airborne benzene in the working environment (0.1 ppm) set by the NIOSH standard [17], was used to classify inhaled airborne benzene into two groups for risk factors analysis: the concentration below 0.05 ppm or lower than 50% of the OEL, and concentrations of 0.05 ppm or higher than 50% of the OEL.

For determination of urinary tt-MA, ten milliliters of urine sample was collected from workers in a sterile container, containing thymol for the preservation of urine, after workers had completed their shift, and sealed and stored at 4 °C until analysis, which took place within one week. The urinary tt-MA analysis followed a standard method from the Bureau of Occupational and Environmental Disease, Department of Disease Control, Ministry of Public Health, Thailand [18]. Urine samples were extracted by solid phase extraction C18-LP 100 mg and eluted with 1% aqueous acetic acid. Urinary tt-MA analysis was performed using high performance liquid chromatography (HPLC) equipped with a ultraviolet (UV) detector operated at 264 nm with the following conditions: the mobile phase of aqueous acetic acid:methanol (82:18); reverse phased C18 column at 20 °C. Urinary tt-MA levels were expressed as microgram per gram creatinine (µg/g Cr). The limit of the detectable level of tt-MA was 0.01 mg/g creatinine or LOD = 10 µg/g Cr. Creatinine (Cr) concentrations (g/mL) in urine samples were determined by spectrophotometry at the same medical laboratory at the university hospital.

This study was approved by Khon Kaen University Ethics Committee in human research (no. HE552240 and HE562237). All participants were given informed consent before entering the study.

### 2.3. Statistical Analysis

Data were analyzed using STATA version 10.0 software (StataCorp, Texus, USA). The association between independent variables and biomonitoring benzene exposure via tt-MA determination was analyzed by bivariate analysis. Factors with a *p*-value of less than 0.20 were selected to be candidate variables in multiple logistic regression models. The confounding factors, which were age and gender, were always included in the multiple logistic regression model. Significant risk factors were screened in a backward stepwise manner using likelihood ratio tests as a selection criterion. The odds ratio (OR) and 95% confident interval of OR (95% CI) for bivariate analysis and the adjusted odds ratio (ORadj) and 95% CI of ORadj for multivariate analysis were presented and a *p*-value < 0.05 indicated a statistical significance of the associated risk factors with a tt-MA detection level from the biomonitoring of benzene exposure.

## 3. Results

### 3.1. Demographic Characteristics of Workers

A total of 170 gasoline station workers, an equal number of females and males, were enrolled in the study (Table 1). The participants ranged in age from 19 to 58 years. The majority of workers were 31–40 years old (35.8%), followed by 21–30 years of age (31.8%). Regarding education level, 61.8% of the participants had attended secondary school, 37.0% had finished primary school, and 1.8% were uneducated. The job function was classified into two groups: fueling workers and cashiers. Fueling workers represented 91.2% of the participants and 8.8% were cashiers. With respect to the workplace zone (location of gasoline station); 37.7% of the participants were from a suburban zone; 36.5% were from an urban zone; and 25.8% were from a rural zone. The rural and suburban zone of stations were grouped as outside the city and in the city, which only included an urban zone.

### 3.2. tt-MA Detection among Benzene Exposed Workers at Gasoline Stations

The urinary tt-MA was detected as a marker of the internal dose of benzene exposure and indicated in the number of workers (n) and proportion (%) of workers in each group (total numbers) of the studied factor, as illustrated in Table 1, Table 2 and Table 3. The results showed that the presence of tt-MA in urine samples was detected in 42 workers (24.7%) with a mean concentration of 74.4 µg/g Cr. The lowest concentration of tt-MA detected was 23.0 µg/g Cr (LOD = 10 µg/g Cr) and the highest was 1127.8 µg/g Cr. Moreover, among the workers positive for tt-MA, there were 11 workers (6.3%) who had urinary tt-MA levels over permissible occupational concentration (500 µg/g Cr), as set by the American Conference of Governmental Industrial Hygienist (ACGIH) [19].

### 3.3. Factors Correlated to Urinary tt-MA Detection

Several significant factors (*p*-value < 0.05) influencing the tt-MA biomarker of the internal dose of benzene were identified by bivariate analysis and included: (a) personal factors (i.e., no other part-time jobs); (b) personal air benzene concentrations found in working environment; and (c) safety practices and training factors (i.e., were close to fuel nozzle while refuelling, no hand washing before eating, and no job training) (Table 1 and Table 2).

From the multivariate analysis, factors (with a *p*-value < 0.20) entered into the multiple logistic regression analysis were the length of employment, a function of working, workplace zone, other part-time jobs, personal benzene concentration, workers who were close to the fuel nozzle during refuelling, job training, and hand washing. After controlling for the confounding effect of age and gender, the adjusted factors significantly associated with the tt-MA biomarker of the internal dose of benzene exposure included no other part-time jobs (ORadj = 4.21, 95% CI: 1.64–10.80); benzene concentration detected in the workplace (ORadj = 10.31, 95% CI: 1.93–55.12); close to the fuel nozzle during refuelling (ORadj = 93.70, 95% CI: 11.07–973.18); and no job training (ORadj = 2.74, 95% CI: 1.11–6.74) (Table 3).

## 4. Discussion

In this study, exposure to benzene was assessed by quantifying urinary tt-MA concentrations, the biomarker of the internal dose of benzene. Results from previous reports have suggested that tt-MA is a reliable biomarker of benzene exposure, especially when exposure levels are low [12,13]. The proportion of gasoline workers with detectable urinary tt-MA at the end of shift work in this study was relatively low (24.7%); however, this percentage was higher than that previously reported for workers exposed to benzene at levels below 1 ppm [11]. Personal exposure to different levels of benzene could influence their tt-MA metabolite release, which has been shown in previous experiments [20]. A range of benzene concentrations from the lowest of 0.006 ppm to the highest of 0.25 ppm correlated with the average tt-MA metabolite as 360 ± 340 µg/g Cr, accounting for 83.6% of benzene exposure [20]. The personal benzene exposure among gasoline station workers found at the lowest range of concentration, which was similar to the recent finding (min:max = 0.01:2.33 ppm), exhibited an excretion of the tt-MA metabolite similar to workers in the shoe industry (57.59 to 1731.38 µg/g Cr) [21] and gasoline station workers in Indonesia (587 µg/g Cr (SD = 1326.5) [22]. The correlations of low benzene concentrations up to 0.05 ppm and the tt-MA metabolite of benzene measured from the benzene exposed workers have not yet clearly been reported. Our analysis could be illustrated by the non-linear regression plotting of the measured tt-MA metabolite and air benzene concentrations, by the significant indication at a *p*-value < 0.001 and the coefficient of Pearson’s correlation r = 0.637, particularly, when the detected tt-MA levels were higher than 500 µg/g Cr, as recommended by the ACGIH [19], these were responding from personal exposure to benzene concentrations of 0.05 ppm or higher.

We found that over 25% of workers excreted tt-MA that exceeded the recommended 500 µg/g Cr. Some previous studies have shown that tt-MA levels in different groups of workers who might be exposed to benzene (e.g., refinery workers [23], turnaround maintenance workers [24], traffic policeman [25,26,27] and gasoline station workers [28]) did not exceed 500 µg/g Cr. In one case study conducted in Thailand, it was found that significant differences in tt-MA levels only existed between workers occupationally exposed to benzene and the general population [29]. The variability in urinary tt-MA levels among workers could also be explained by different competing routes of benzene metabolism that compete for muconic acid and other metabolites requiring GSTT (glutathione-S-/sulfo- transferases) activity. In addition, CYP2E1 (cytochrome P4502E1), which is a key metabolic step, could be reducible of the tt-MA metabolite by alcohol consumption. This had been previously confirmed by epidemiological studies among benzene handling workers in a refinery [30,31]. However, the urinary concentration of tt-MA in excess of those recommended by the ACGIH was identified in some gasoline station workers exposed to relatively low levels of air benzene (<0.1 ppm) in this study, then tt-MA may represent an important method for assessing benzene exposure at gasoline stations. This supports the fact that tt-MA is a sensitive biomarker for benzene exposure as previously reported [24]. Although some studies have proposed that it may not be a reliable biomarker of exposure to low benzene concentration, it was still confirmed as a relatively convenient biomarker of urinary benzene metabolite for screening purposes by cost-efficient analysis [25,32]. However, cigarette smoking and alcohol drinking should be controlled as the confounding factor of selecting the benzene metabolite as the biomarker of exposure to benzene. Although it was pointed out that the intake of sorbic acid preserved food altered the background tt-MA excretion [33], our result did not find a significant effect on levels of the detected tt-MA. However, consumption of preserved foods containing sorbic acid could be also further minimized for specific tt-MA use in the biological monitoring of occupational exposure to benzene following the previous suggestion [33].

An association between the urinary tt-MA biomarker of the internal dose of benzene exposure and personal air benzene concentrations found in this study from the multivariate analysis supports a previous study of gasoline station workers in one Thai city [34]. The results show that based strictly on personal air monitoring measurements, working under an air benzene concentration at 0.05 ppm or higher than 0.05 ppm indicated a higher detection of urinary tt-MA of workers working under an air benzene concentration less than 0.05 ppm. That setting of 0.05 ppm was compared to 10% of the occupational exposure limit (0.5 ppm) set by ACGIH [19] and 50% of the recommended exposure limit (0.1 ppm) of using an 8 h time-weighted average (TWA) set by NIOSH [17]. This is a condition that the gasoline workers should be aware of protecting themselves against even though the ambient benzene concentration was below the permissible exposure level. The previous study also showed that workers could exceed acceptable health risk levels with chronic exposure to benzene at the concentration of 10% of the permissible exposure level set by ACGIH [35,36].

Safety practices were found to be an important determinant that affected occupational exposure to benzene. This study found that the majority of workers that dispensed fuel had a propensity to work close to the fuel nozzle during refueling. Moreover, multiple logistic regression analyses indicated that workers who closely observed the fuel nozzle had a higher risk of the tt-MA biomarker detecting the internal dose benzene, when compared to workers that did not exhibit this behavior. This is in agreement with a previous study showing that the fueling workers had the highest health risk of benzene exposure [35] and that inhalation was the primary route of benzene exposure in the occupational setting [37]. Therefore, behavior identified the risk for the fueling workers to directly inhale fuel vapors containing benzene. In addition, those workers were also exposed to benzene from ambient air in the work setting. A general safety practice, specifically hand washing, was also found to be a significant factor contributing to the non-detection of benzene exposure. The univariate analysis showed that workers who did not wash their hands before eating had a significantly higher risk of benzene exposure via the oral route than the workers that washed their hands. Our previous study suggested that gasoline station workers exposed to benzene were potentially exposed via the oral route while eating at work [36]. Furthermore, another significant risk factor was the absence of job training. The adjusted association analysis showed that workers without job training had a higher risk of benzene exposure through the tt-MA detection marker than those workers receiving job training. This finding strongly suggests a need for worker safety training to protect workers from occupational benzene exposure.

The last factor was no other part-time jobs. The multivariate analysis indicated that workers who had no other part-time jobs had a higher risk of tt-MA detection as the benzene internal dose of exposure when compared to those that had other part-time jobs. The explanation might be because it is characteristic of workers who had no other part-time jobs to work more hours or more days per week at the gasoline station. Collectively, our study found that 73.8% of those workers positive for tt-MA levels worked in a rural and a suburban zone where they provided a higher amount of daily service than an urban zone [9]. Those workers could potentially experience high exposure levels of benzene in the work environment at gasoline stations where benzene concentrations were found to be two-times higher than the concentration found in traffic areas [9].

## 5. Conclusions

Benzene exposure determined by the urinary tt-MA biomarker of internal dose of benzene among workers at gasoline stations was indicated in 24.7% of workers with a tt-MA concentration ranging from 23.0 to 1127.8 µg/g Cr. In addition, 26.2% of these workers had urinary tt-MA concentrations that exceeded the recommended safety level (>500 µg/g Cr). The multiple logistic regression analysis confirmed an association of personal air benzene concentration with the urinary tt-MA biomarker of the internal dose of benzene exposure among gasoline station workers. Workers who were close to the fuel nozzle while refuelling had a significantly higher risk of benzene exposure compared to those who did not exhibit this behavior. Furthermore, the study revealed that gasoline station workers with no other part-time jobs or no job training had higher a potential risk of tt-MA detection as a marker of benzene internal dose than those who had part-time jobs and job training. Collectively, our findings suggest that employers should provide essential biological monitoring with compliance to a biomarker and health surveillance program. Work safety training for gasoline station workers is an important issue to minimize benzene exposure. Hence, if there are air benzene concentrations at gasoline stations lower than OEL-ACGIH (0.5 ppm), efforts should be made in Thailand to reduce the permissible exposure limit of benzene exposure level to lower than that currently set by the NIOSH (0.1 ppm), or to set the reference of the occupational action concentration to 0.05 ppm as found in this study, in a health surveillance program of benzene exposed workers at gasoline stations or other similar workplaces.

## Figures and Tables

**Table 1:** Bivariate analysis of the relationship between personal factors and tt-MA detection among the 170 gasoline station workers.

Factor	Total Workers	tt-MA Detected Workers n (%)	OR	95% CI	*p*-Value
**Gender**					
Male	85	23 (27.1)	1.29	0.64–2.59	0.477
Female	85	19 (22.4)	1.00		
**Age**					
≥30 years	101	29 (28.7)	1.74	0.83–3.64	0.138
<30 years	69	13 (18.8)	1.00		
**Education**					
Primary school and lower	65	17 (26.2)	1.13	0.56–2.31	0.731
Secondary school and higher	105	25 (23.8)	1.00		
**Length of employment**					
≥1 year	114	32 (28.1)	1.80	0.81–3.98	0.150
<1 year	56	10 (17.9)	1.00		
**Function of working**					
Fueling worker	155	36 (23.2)	0.45	0.15–1.36	0.158
Cashier	15	6 (40.0)	1.00		
**Working day**					
6 days/week	136	34 (25.0)	1.08	0.45–2.62	0.859
7 days/week	34	8 (23.5)	1.00		
**Workplace zone**					
Outside the city (Rural and Suburban)	108	31 (28.7)	1.87	0.86–4.04	0.114
In the city (Urban)	62	11 (17.7)	1.00		
**Other part** **-** **time jobs**					
No	70	27 (38.6)	3.56	1.71–7.38	0.001 ^1^
Yes	100	15 (15.0)	1.00		
**Close to smoker**					
Yes	98	22 (22.4)	0.75	0.37–1.52	0.427
No	72	20 (27.8)	1.00		
**Eating food** **-** **contained sorbic acid**					
Yes	159	38 (23.9)	0.55	0.15–1.98	0.360
No	11	4 (36.4)	1.00		
**Annual health check-up**					
No	155	40 (25.8)	2.26	0.49–10.46	0.297
Yes	15	2 (13.3)	1.00		

^1^ Significant association at *p*-value < 0.05; *n* = number of workers had tt-MA detection (23.0–1127.8 µg/g Cr).

**Table 2:** Bivariate analysis of the relationship between factors of work characteristics and environment, safety practices and training, and tt-MA detection among the 170 gasoline station workers.

Factors	Total Workers	tt-MA Detected Workers n (%)	OR	95% CI	*p*-Value
**Work characteristics and environment**					
**Direct skin contact with gasoline**					
Yes	152	36 (23.7)	0.62	0.22–1.77	0.384
No	18	6 (33.3)	1.00		
**Personal benzene concentration**					
≥0.05 ppm	40	16 (40.0)	2.67	1.24–5.73	0.010 ^1^
<0.05 ppm	130	26 (20.0)	1.00		
**Safety practices and training**					
**Eating snack or drinking water in the workplace**					
Yes	159	38 (23.9)	0.55	0.15–1.98	0.373
No	11	4 (36.4)	1.00		
**Having lunch in the workplace**					
Yes	154	37 (24.0)	0.70	0.23–2.13	0.533
No	16	5 (31.2)	1.00		
**Close to fuel nozzle during refuelling**					
Yes	140	40 (28.6)	5.60	1.27–24.62	0.023 ^1^
No	30	2 (6.7)	1.00		
**Hand washing**					
No	8	5 (62.5)	5.63	1.28–24.67	0.022 ^1^
Yes	162	37 (22.8)	1.00		
**PPE using**					
No	95	25 (26.3)	1.22	0.60–2.47	0.583
Yes	75	17 (22.7)	1.00		
**Job training**					
No	91	30 (33.0)	2.74	1.29–5.84	0.009 ^1^
Yes	79	12 (15.2)	1.00		

^1^ Significant association at *p*-value < 0.05; *n* = number of workers had tt-MA detection (23.0–1127.8 µg/g Cr).

**Table 3:** Multiple logistic regression analysis of potential risk factors and urinary tt-MA detection among the 170 gasoline station workers.

Factors	Total Workers	tt-MA Detected Workers n (%)	OR	OR_adj_	95% CI of OR_adj_	*p*-Value
**Gender**						
Male	85	23 (27.1)	1.29	1.55	0.63–3.78	0.338
Female	85	19 (22.4)	1.00	1.00		
**Age**						
≥30 years	65	17 (26.2)	1.13	1.49	0.59–3.75	0.396
<30 years	105	25 (23.8)	1.00	1.00		
**Other part** **-** **time jobs**						
No	70	27 (38.6)	3.56	4.21	1.64–10.80	0.003 ^1^
Yes	100	15 (15.0)	1.00	1.00		
**Close to fuel nozzle during refuelling**						
Yes	140	40 (28.6)	5.60	93.70	11.07–793.18	<0.001 ^1^
No	30	2 (6.7)	1.00	1.00		
**Personal benzene concentration**						
≥0.05 ppm	40	16 (40.0)	2.67	10.31	1.93–55.12	0.006 ^1^
<0.05 ppm	130	26 (20.0)	1.00	1.00		
**Job training**						
No	91	30 (33.0)	2.76	2.74	1.11–6.74	0.028 ^1^
Yes	79	12 (15.2)	1.00	1.00		
**Hand washing**						
No	8	5 (62.5)	5.63	4.55	0.45–45.49	0.198
Yes	162	37 (22.8)	1.00	1.00		

^1^ Significant association at *p*-value < 0.05; *n* = number of workers had tt-MA detection (23.0–1127.8 µg/g Cr).
